# ReptTraits: a comprehensive dataset of ecological traits in reptiles

**DOI:** 10.1038/s41597-024-03079-5

**Published:** 2024-02-27

**Authors:** Oleksandra Oskyrko, Chunrong Mi, Shai Meiri, Weiguo Du

**Affiliations:** 1 Key Laboratory of Animal Ecology and Conservation Biology, Institute of Zoology, Chinese Academy of Sciences, Beijing, China; 2 University of Chinese Academy of Sciences, Beijing, China; 3 School of Zoology & the Steinhardt Museum of Natural History, Tel Aviv University, Tel Aviv, Israel

**Keywords:** Herpetology, Macroecology

## Abstract

Trait datasets are increasingly being used in studies investigating eco-evolutionary theory and global conservation initiatives. Reptiles are emerging as a key group for studying these questions because their traits are crucial for understanding the ability of animals to cope with environmental changes and their contributions to ecosystem processes. We collected data from earlier databases, and the primary literature to create an up-to-date dataset of reptilian traits, encompassing 40 traits from 12060 species of reptiles (Archelosauria: Crocodylia and Testudines, Rhynchocephalia, and Squamata: Amphisbaenia, Sauria, and Serpentes). The data were gathered from 1288 sources published between 1820 and 2023. The dataset includes morphological, physiological, behavioral, and life history traits, as well as information on the availability of genetic data, IUCN Red List assessments, and population trends.

## Background & Summary

Species traits are fundamental to macroecological and macroevolutionary investigations. Trait datasets allow for integrating a diverse range of physiological, ecological, morphological, and life history data to explore organismal ecology and evolution^1–4^. Comparative studies regularly use trait data to study topics such as animal physiology, ecology, and behaviour. These studies have a rich history of aggregating extensive trait datasets and examining diverse hypotheses at the species level. Analyses may focus, for example, on factors influencing rapid morphological diversification, or the role of divergent adaptation in speciation^5–8^. The consolidation of trait data into comprehensive databases enhances ongoing research efforts by centralizing scattered information into a unified repository. Unrestricted access to such a repository has the potential to significantly streamline future investigations into animal diversity, ecology, evolution, and conservation.

Reptiles are a highly interesting group of animals that demonstrate a strong sensitivity to environmental factors, including temperature, precipitation, landscape features, and soils^9–14^. A growing body of literature describes the physiological, morphological, and performance traits of reptiles^15–19^, contributing to global analyses that aim to enhance our understanding of the life history and evolution of these creatures. Recently, Meiri^20^ published a comprehensive database containing basic physiological and ecological traits for lizards. However, this database excludes other groups of reptiles, such as archelosaurs (crocodiles and turtles), Rhynchocephalia (the tuatara), and other squamates (i.e. snakes). While ecological databases have been published for turtles, snakes, and crocodiles over the past decades, they are often small and limited to specific species or countries^21–24^.

Therefore, we have compiled a database summarizing a vast amount of data for all reptiles, including amphisbaenians, lizards, crocodiles, snakes, and turtles. This database is designed to be user-friendly and easily updated as the literature grows. In this article, we present this dataset, which contains ecological and physiological traits of all reptile species (Archelosauria: Crocodylia and Testudines, and Lepidosauria: Rhynchocephalia, and Squamata: Amphisbaenia, Sauria, and Serpentes). We collected data for 40 traits central to many ecological questions. For most traits, we incorporated lizard data from Meiri^20^ published database and added new data not covered in that dataset from various literature sources. Our data collection involved published and online databases, as well as primary and secondary literature^25–27^. The Reptile Database^28^ served as our taxonomic backbone. We anticipate that making this data available will reveal both gaps and errors inherent in compiling a dataset of this size, enabling efforts to address these shortcomings.

## Methods

We compiled a dataset for 12060 species following the taxonomy in the Reptile Database^28^, including 40 physiological, morphological, ecological and behavioural traits, and habitat variables (Table S1). We divided traits into six categories: habitat; behaviour; morphology; ife history; physiology; and conservation (see Fig. 2). We collected data for four reptile Orders: Crocodylia (alligators and crocodiles, n = 27 species), Testudines (turtles and tortoises, n = 361 species); Rhynchocephalia (tuatara, n = 1 species), and Squamata, comprised of three Sub-Orders: Sauria (lizards, n = 7415 species), Amphisbaenia (n = 202 species), and Serpentes (snakes, n = 4073 species). We used the “one-row-per-species” format because information on within-species variation is very limited for most species. Our dataset compilation consisted of several steps. First, we identified sources of trait data. Second, we manually extracted the data and transcribed them into a comma-separated values (CSV) file (a “raw” data file) and retained the measurement units as published. Then, we read all raw data files, checked data quality and combined them into a single Excel file of standardised observations and units of measurement. Finally, we performed additional data quality checks on the standardised observations, correcting processing errors and checking for additional issues (see Figure S1).

We searched for additional species-specific data in literature published between 1974 and 2023 using Google Scholar (https://scholar.google.com) and Web of Science (https://www.webofscience.com). First, we searched published databases. In the search phrases, we combined taxon names (Class or Order) with one of the following keywords: reptil*, squamat*, lizard*, snake*, turtle*, testudin*, tortois*, tuatara*, crocodil*, alligator*. In total, we used 16 published databases and four online databases^25–27^. In addition, we considered all citations in published databases and major reviews and included any additional papers. We have added primary sources from published databases for sources of individual trait values. If primary sources were not listed for a species, we cited only the database. We searched separately for some species of reptiles that had unclear information. In Google Scholar and Web of Science we searched for the species name (e.g., Ablepharus alaicus*”). We focused on Crocodylia, Testudines and Serpentes species because most of the data for Sauria and Amphisbaenia were published in Meiri^20^. However, if new information for lizards was found during this search, these data also were added to our dataset. We added the higher-level taxonomic classification (family, order) following the Reptile Database^28^. If a species with data could not be identified automatically, we corrected the entry manually after searching for relevant synonyms in the reptile database^28^. We contacted the Reptile Database team and received permission for using their data under an open license from the original data generators. We translated sources from languages other than English when possible. Papers that were inaccessible, or written in languages we could not translate, were excluded. Our review involved examining approximately 2000 sources from primary scientific literature, books, public journals, and online resources. Reviews and meta-analyses guided the selection of appropriate papers for extracting original data. After reading the title and abstract of each article, we decided whether to read the entire article and extract data from it, based on whether the paper reported species-specific information on the ecological traits we were trying to find (see Figure S1). We focused on papers that provided species-specific information on the ecological traits under investigation. From 1288 of the 2000 sources we extracted data. Data extraction involved reviewing text, online supplementary materials, or tables within each source. For species represented in multiple rows (e.g., appearing with different names, subspecies, or data sources), we consolidated the information into a single row (see details below). This approach aimed to present a unified representation of consensus data for each species. The phylogenetic tree was drawn using “ggtree” package^29,30^ for all species of the Class Reptilia^31–33^.

## Data Records

We amassed data for a total of 12060 species, belonging to 1255 genera, 92 families, and four orders (included six major groups: the orders Testudines, Crocodilia, and Rhynchocephalia and the three sub-orders of Squamata: Amphisbaenia, Sauria, and Serpentes). We include 1288 data sources in the dataset, with 1284 sources coming from published scientific literature (including books and published databases) and four online databases^25–27^. Missing data were coded as ‘NA’. We created an excel spreadsheet containing both the dataset content and the column descriptions as separate worksheets (Table 1: individual trait values; Table 2: sources of individual trait values.; Table 3, citations; Table 4, trait definitions). The dataset is provided as an Excel file named ReptTraits dataset v1-1.xlsx in the Figshare repository^34^.

Our dataset includes eight types of taxonomic data and metadata, and 40 traits as follows: Species, Order, Suborder, Family, Genus, Description author/s, Description year, Subspecies, main biogeographic region, microhabitat, habitat type, minimal and maximal elevation, mean annual temperature, temperature seasonality, precipitation seasonality, insular/endemic, venomous, diet, active time, dorsal colour and pattern, foraging mode, pupil shape, fangs (front-fanged, non-fanged or rear-fanged), maximum longevity, maximum body mass and maximum length (TL, SVL and SCL), hatchling/neonate mass, reproductive mode, sex-determining mechanism (GSD or TSD), mean number of offspring per litter or number of eggs per clutch, smallest and largest clutch size, number of litters or clutches produced per year, egg length and width, mean, minimum and maximum body temperature (Tb) (in the field), genetic data (whether these exist on GenBank or not), IUCN red list assessments, and IUCN population trends (e.g., Table S1). We present each type of data in a column, or set of columns, that can be instantly used for analyses. We collected length data separately for males, females, juvenile and unsexed individuals. Most mass data are based on lengths, transformed to masses based on taxon-specific equations (accounting for the degree of limb loss in relevant lineages)^15,35^. We used only maximum values for longevity, body mass, Total length, snout vent length (SVL), and carapace length (CL; of turtles). For some traits, we average the minimum and maximum reported means values (e.g., clutch size, body size, Tb). We collected “Maximum length SVL”, “Maximum female SVL”, “Maximum male SVL” or “Maximum juvenile SVL” data for Crocodylia, Rhynchocephalia, and Squamata. Testudine measurements of the same columns have different meanings: they are Carapace lengths. If we had more than one mean for a specific trait for a given species, we averaged the smallest and highest reported means. When means were unavailable, we averaged the minimum and maximum reported values^20^. Unfortunately, due to the lack of data, we could not collect the maximum, minimum and average values for all traits, so some traits have only the maximum or average values Our dataset can easily be reproduced, updated, and expanded to include a wider range of species, other taxa or traits.

## Technical Validation

We thoroughly examined the dataset to ensure variable consistency, including accurate species and trait naming. We assessed data integrity by identifying outliers and verifying correct data types and consistent use of units. We updated species binomials according to the latest version of the reptile database^28^. Data sources that posed challenges in interpretation, lacked extractable raw data, or relied on data imputation, were excluded from the dataset (see Figure S1).

Quality control measures included generating plots and scrutinizing them for outliers. When we identified issues during standardization or quality control, we first verified whether they stemmed from transcription errors by comparing the raw file to the source data. If this was the case, we corrected the data. Otherwise, we investigated whether the problem originated from a standardization step failure, such as a programming error, and rectified the standardization scripts accordingly. In cases where an error persisted, we examined the source paper for potential issues, such as incorrect units or misplaced decimal points. In those few cases when inconsistencies occurred, we decided on a solution based on double-checking the original sources and mutual agreement between the first, second and last authors. If no solution was found, we deleted the datum.

## Usage Notes

**Fig. 1:**
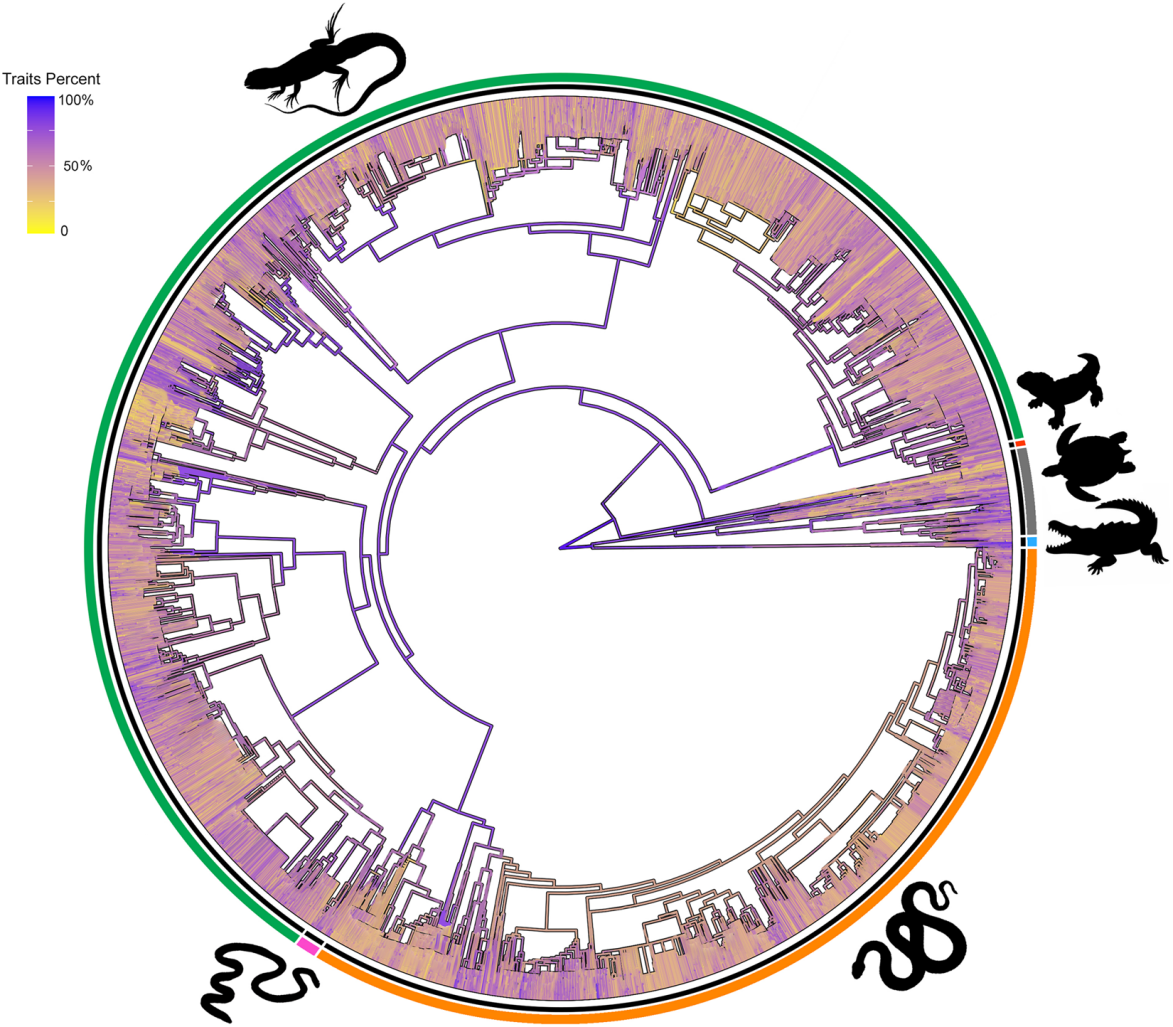
Distribution of percentage of traits with data for each species across the phylogeny of all reptiles. Each of the six major groups are represented in the dataset: pink = Amphisbaenia, blue = Crocodilia, red = Rhynocephalia (tuatara), green = Sauria, orange = Serpentes, and grey = Testudines.

**Fig. 2:**
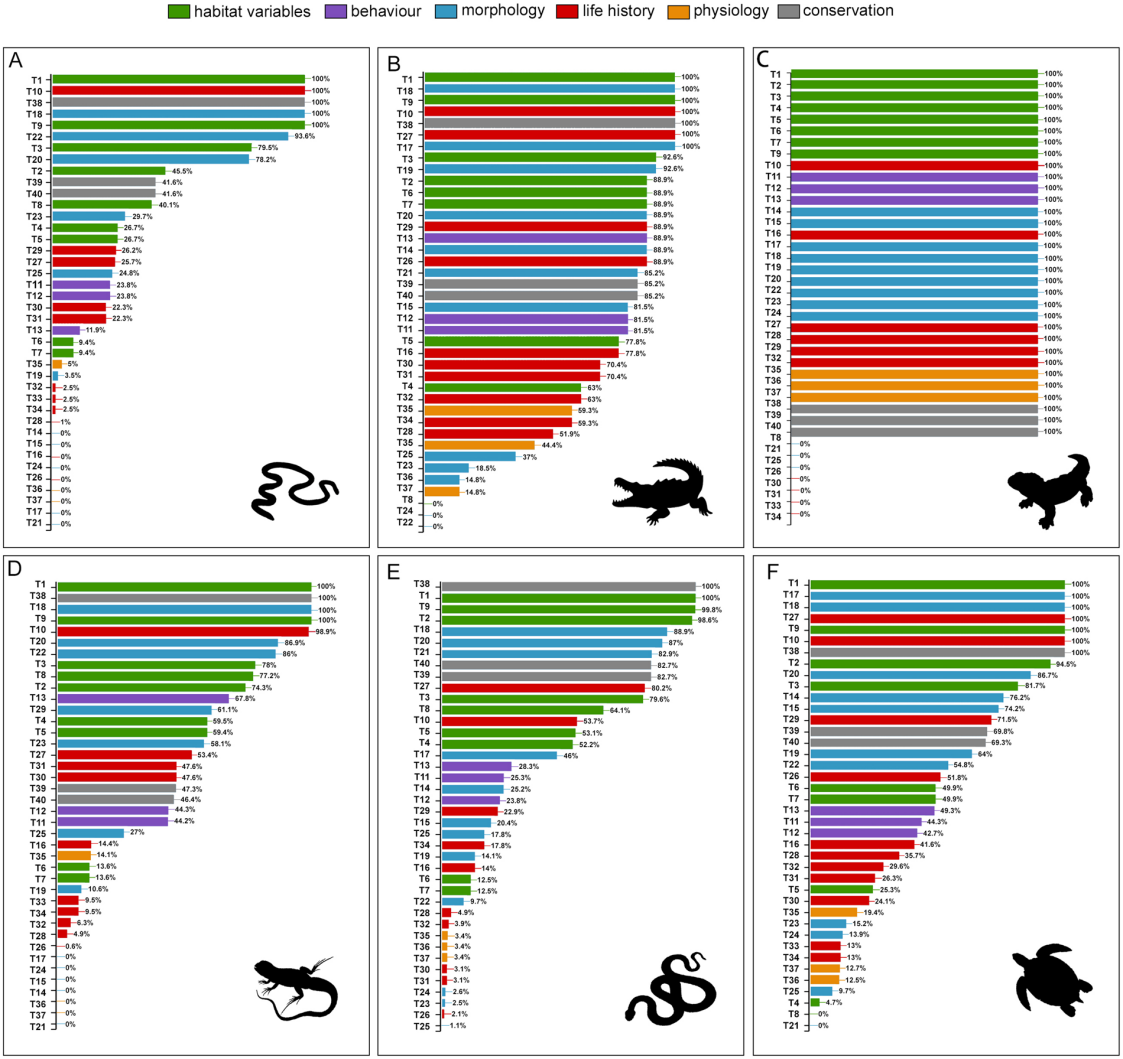
Percentage of species collected for each trait for all reptiles. Data were obtained from the present study dataset (amphisbaenians, crocodiles, lizards, snakes, tuatara and turtles). A - data for Amphisbaenia, B - data for Crocodilia, C - data for Rhynocephalia, D - data for Sauria, E - data for Serpentes and F - data for Testudines. The names of the traits (T1-T40) follow those in Table S1. Colours represent trait categories: green: habitat variables; purple: behaviour; blue: morphology; red: life history; orange: physiology; grey: conservation.

Compared with previously published databases^21–24^ our dataset is bigger (greater number of species and traits), thereby advancing the current state of knowledge in the field. However, we acknowledge certain limitations inherent in our dataset (and others), including taxonomic (Figs. 1, 2) and geographic biases in sampling. The data gaps identified in our study are regrettably common (Figs. 1, 2) and pertain to taxa (e.g., Amphisbaenia, Dibamidae) that are notably rare or challenging to study (‘Linnean Shortfall’) due to biological constraints (e.g., fossoriality). Similarly, the faunas of some regions are difficult to access or study (e.g., in certain war-torn regions and regions with poor transportation infrastructure; ‘Wallacean Shortfall’). Additionally, some gaps are attributed to difficulties in accessing scientific literature, particularly due to language barriers and related citation indexing challenges^36^.

The dataset can also easily be expanded and corrected if errors are identified. We encourage researchers to let us know if they find any error in our dataset or if they publish new data that should be included in future versions. Users can utilize the supplied data to compile and standardise the dataset with different standardisation parameters or output units. The data descriptor was peer reviewed in 2023 based on the data available on the platform at the time.

### Supplementary information

Supplementary Information Table S1

Supplementary Information Figure S1

## Data Availability

Clarification of workflow to create our dataset is available as Supplementary Figure S1. Name and definition of traits are presented in Table S1. Additionally, our dataset is provided at FigShare^34^.
