# Supplementary material for: ReptTraits: a comprehensive dataset of ecological traits in reptiles
Source: Sci Data. 2024 Feb 27;11:243. doi: 10.1038/s41597-024-03079-5 (PMC10899194; doi:10.1038/s41597-024-03079-5)
Supplement: Supplementary file 1 — Supplementary Information Table S1 [file 41597_2024_3079_MOESM1_ESM.pdf]

ReptTraits: a comprehensive dataset of ecological traits in reptiles

Oleksandra Oskyrko, Chunrong Mi, Shai Meiri, Weiguo Du

Table S1. Name and definition of traits used in the dataset for Amphisbaenia, Crocodilia, Rhynocephalia, Sauria, Serpentes and Testudines.

Number	Name	Description
T1	Main biogeographic region	Realm designations are from Falschi et al. (2023) and GARD 1.7 ranges (Caetano et al. 2022).
T2	Microhabitat	Whether a species is arboreal, fossorial or cryptic (i.e., almost always only found under objects such as rocks or logs), saxicolous, semi-aquatic, terrestrial or a combination of some of those.
T3	Habitat type	Different kind of habitat, including grasslands, swamps, forests, deserts, oceans, and other arid environments were distribution reptiles. Following IUCN (2023) and primary literature (for species that do not have information in IUCN).
T4	Minimal elevation (m)	Minimal elevation at which the species was observed (meters above sea level).
T5	Maximum elevation (m)	Maximal elevation at which the species was observed (meters above sea level).
T6	Mean Annual Temperature (°C)	Mean annual temperature (°C; Roll et al. 2017, Karger et al. 2017, Stark et al. 2018, Meiri et al. 2020).
T7	Temperature Seasonality (standard deviation*100)	Temperature seasonality (standard deviation × 100; Roll et al. 2017, Karger et al. 2017, Stark et al. 2018).
T8	Seasonality Precipitation (coefficient of variation × 100)	Precipitation seasonality (coefficient of variation × 100; Roll et al. 2017, Karger et al. 2017, Stark et al. 2018, Zimin et al. 2022).

T9	Insular/endemic (yes or no)	Whether species is endemic or reside only on islands.
T10	Venomous (yes or no)	The species is venomous or not.
T11	Diet	Species is herbivorous (consumes mostly plants), carnivorous (eats only animal matter), or omnivorous (eats animal and plant matter.
T12	Diet: comments	Explained which groups of animals the species can eat.
T13	Active time	Whether a species is diurnal, nocturnal, cathemeral, crepuscular or polyphasic.
T14	Dorsal colour	Dorsal colour of reptiles.
T15	Dorsal pattern	The presence or absence of a pattern on the back of reptiles.
T16	Foraging mode	Whether the species is reported to be an ambush predator (“sit and wait”; AMB), an active forager (“Active foraging”; ACT) or uses a mixed strategy (“mixed”; ACT/AMB).
T17	Pupil Shape	The shape of the pupil in animals.
T18	Fangs	The presence of fangs or their shape.
T19	Maximum Longevity (years)	Longevity data are the maximum age (in years) reported for each species from the literature.
T20	Maximum body mass (g)	The maximum body mass of grams (without sex).
T21	Maximum total length ("TL", mm)	Total length in millimetres
T22	Maximum length ("SVL", mm)/straight carapace length for turtles ("SCL", mm)	We collected "Maximum length" for Crocodylia, Rhynchocephalia, Squamata and Amphisbaenia. But Testudines included in the same colum but different measure "straight carapace length for turtles".
T23	Maximum female length ("SVL", mm)/straight carapace length for turtles ("SCL", mm)	We collected "Maximum female length" for Crocodylia, Rhynchocephalia, Squamata and Amphisbaenia. But Testudines included in the same colum but different measure "straight carapace length for turtles".
T24	Maximum male length ("SVL", mm)/straight carapace length for turtles ("SCL", mm)	We collected "Maximum male length" for Crocodylia, Rhynchocephalia, Squamata and Amphisbaenia. But Testudines included in the same colum but different measure "straight carapace length for turtles".
T25	Maximum juvenile length ("SVL", mm)/straight	We collected "Mean juvenile length" for Crocodylia, Rhynchocephalia, Squamata and Amphisbaenia. But Testudines

	carapace length for turtles ("SCL", mm)	included in the same colum but different measure "straight carapace length for turtles".
T26	Hatchling/neonate mass (g)	Weight of hatchling or neonate.
T27	Reproductive mode	Whether a species is oviparous or viviparous, or whether there are reports on both parity modes “mixed” (ovoviviparous).
T28	Sex-determining mechanism (GSD or TSD)	The sex-determining system in which the two sexes have different sex chromosomes. There are two types of sex-determining system: GSD (genotypic sex determination) and TSD (temperature-dependent sex determination).
T29	Mean number of offspring per litter or number of eggs per clutch	Means of clutch or litter sizes.
T30	Smallest clutch size	Minima (lowest reported means) of clutch or litter sizes.
T31	Largest clutch size	Maxima (highest reported means) of clutch or litter sizes.
T32	Number of litters or clutches produced per year	For Number of litters or clutches produced per year, we use species means, if available, or midpoints (e.g. the average between the largest and smallest known clutches) if means are not reported. Similarly, we use a midpoint of the largest and smallest mean if multiple means were reported.
T33	Egg length (mm)	The number of millimetres in length of the egg (if present).
T34	Egg width (mm)	The number of millimetres in width of the egg (if present).
T35	Mean Tb	The mean reported mean body temperatures of animal (in °C).
T36	Minimum Tb	We collected data "minimum mean Tb" for lizards according to Meiri (2018). For other groups of reptiles (Crocodylia, Rhynchocephalia, Serpentes and Testudines), we collected only "minimum Tb".
T37	Maximum Tb	We collected data "maximum mean Tb" for lizards according to Meiri (2018). For other groups of reptiles (Crocodylia, Rhynchocephalia, Serpentes and Testudines), we collected only "maximum Tb".

T38	Genetic data (yes or not)	Whether there are published accounts of the genetic data of the species (GenBank records), and the genes used to infer it.
T39	IUCN redlist assessment	IUCN red – list assessment (IUCN 2023).
T40	IUCN population trend	IUCN population trends (IUCN 2023).

References

Caetano, G. H. O. et al. Automated assessment reveals that the extinction risk of reptiles is widely underestimated across space and phylogeny. PLOS Biol. 20(5), e3001544 (2022).

Falaschi, M. et al. Global bioregions of reptiles confirm the consistency of bioregionalization processes across vertebrate clades. Glob. Ecol. Biogeogr. 32(8), 1272–1284 (2023).

IUCN. The IUCN Red List of Threatened Species. https://www.iucnredlist.org (2023).

Karger, D. N. et al. Climatologies at high resolution for the Earth's land surface areas. Sci. Data 4, 170122 (2017).

Meiri, S. Traits of lizards of the world: Variation around a successful evolutionary design. Glob. Ecol. Biogeogr. 27(10), 1–5 (2018).

Meiri, S., Feldman, A., Schwarz, R. & Shine, R. Viviparity does not affect the numbers and sizes of reptile offspring. J. Anim. Ecol. 89(2), 360–369 (2020).

Roll, U. et al. The global distribution of tetrapods reveals a need for targeted reptile conservation. Nat. Ecol. Evol. 1, 1677–1682 (2017).

Stark, G., Tamar, K., Itescu, Y., Feldman, A. & Meiri, S. Cold and isolated ectotherms: drivers of reptilian longevity. Biol. J. Linn. Soc. 125(4), 730–740 (2018).

Zimin, A. et al. A global analysis of viviparity in squamates highlights its prevalence in cold climates. Glob. Ecol. Biogeogr. 31, 2437–2452 (2022).
