# Supplementary material for: ReptTraits: a comprehensive dataset of ecological traits in reptiles
Source: Sci Data. 2024 Feb 27;11:243. doi: 10.1038/s41597-024-03079-5 (PMC10899194; doi:10.1038/s41597-024-03079-5)
Supplement: Supplementary file 2 — Supplementary Information Figure S1 [file 41597_2024_3079_MOESM2_ESM.pdf]

ReptTraits: a comprehensive dataset of ecological traits in reptiles

Oleksandra Oskyrko, Chunrong Mi, Shai Meiri, Weiguo Du

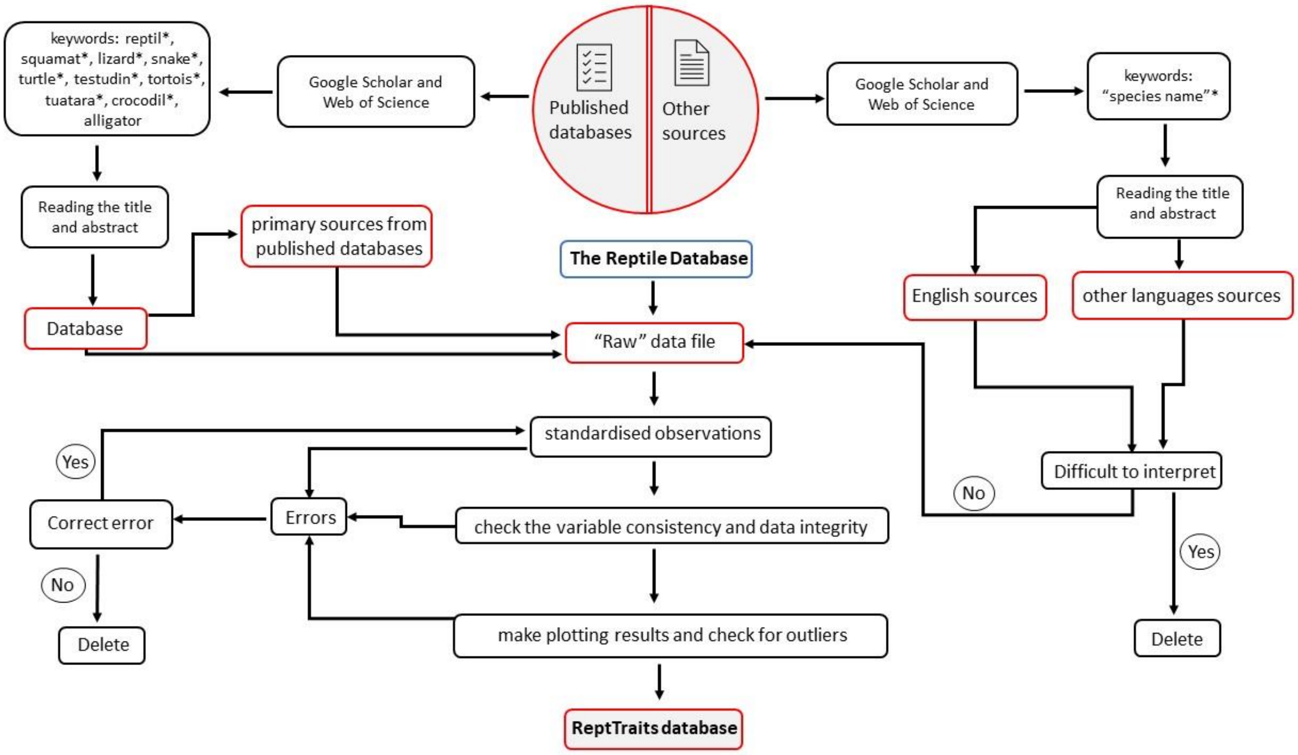

Fig. S1. Clarification of workflow to create dataset
